# Predicting miRNA-Disease Associations by Incorporating Projections in Low-Dimensional Space and Local Topological Information

**DOI:** 10.3390/genes10090685

**Published:** 2019-09-06

**Authors:** Ping Xuan, Yan Zhang, Tiangang Zhang, Lingling Li, Lianfeng Zhao

**Affiliations:** 1 School of Computer Science and Technology, Heilongjiang University, Harbin 150080, China; 2 School of Mathematical Science, Heilongjiang University, Harbin 150080, China

**Keywords:** miRNA-disease associations, non-negative matrix factorization, graph regularization, projection of miRNAs and diseases, sparse characteristic of associations

## Abstract

Predicting the potential microRNA (miRNA) candidates associated with a disease helps in exploring the mechanisms of disease development. Most recent approaches have utilized heterogeneous information about miRNAs and diseases, including miRNA similarities, disease similarities, and miRNA-disease associations. However, these methods do not utilize the projections of miRNAs and diseases in a low-dimensional space. Thus, it is necessary to develop a method that can utilize the effective information in the low-dimensional space to predict potential disease-related miRNA candidates. We proposed a method based on non-negative matrix factorization, named DMAPred, to predict potential miRNA-disease associations. DMAPred exploits the similarities and associations of diseases and miRNAs, and it integrates local topological information of the miRNA network. The likelihood that a miRNA is associated with a disease also depends on their projections in low-dimensional space. Therefore, we project miRNAs and diseases into low-dimensional feature space to yield their low-dimensional and dense feature representations. Moreover, the sparse characteristic of miRNA-disease associations was introduced to make our predictive model more credible. DMAPred achieved superior performance for 15 well-characterized diseases with AUCs (area under the receiver operating characteristic curve) ranging from 0.860 to 0.973 and AUPRs (area under the precision-recall curve) ranging from 0.118 to 0.761. In addition, case studies on breast, prostatic, and lung neoplasms demonstrated the ability of DMAPred to discover potential disease-related miRNAs.

## 1. Introduction

Several studies have shown that the abnormal expression of microRNAs (miRNAs) is inextricably related to the occurrence and development of diseases [1,2,3,4,5]. As the number of identified miRNAs continues to increase, a large number of disease-related miRNAs (disease miRNAs) are waiting to be identified.

Some of the methods previously used to predict diseases-associated miRNAs can be divided into two categories. The first category includes the use of regulatory relationships between miRNAs and their target genes to predict potential associations between the miRNA and the disease [6]. Since the number of experimentally validated target genes is not sufficient, some predictive algorithms such as PITA [7], TargetScan [8], and MiRanda [9] are needed to extrapolate the existence of target gene-miRNA associations [10,11,12,13]. The likelihood of a miRNA associated with a disease is predicted based on the similarity or interaction between disease-related target genes and miRNA-related target genes. Since the predictions from such methods have higher false positives, these methods have limited applicability.

Another category of methods is based on the notion that miRNAs with similar functions are often associated with similar diseases [14,15,16,17], and thus, these methods do not depend on the interaction between a miRNA and its corresponding target genes. First, the functional similarity between miRNAs was calculated by the miRNA-related diseases [18]. These methods constructed a miRNA network according to the miRNA functional similarity, and conducted random walks on the miRNA network [19,20] or used information from neighboring nodes [21]. However, such methods rely on a group of seed miRNAs associated with the disease and cannot be applied to new diseases. Some methods have been improved in this regard. They established heterogeneous networks by employing disease similarities, miRNA similarities, and known associations between diseases and miRNAs. Global random walks [22,23], matrix completion [16], or matrix factorization methods [24,25,26,27,28] based on heterogeneous networks are used to predict the association score between miRNA and disease. There are some methods that use path-based search algorithms [29,30] and machine learning methods [31,32,33] for association prediction.

In this study, we propose an effective method, DMAPred, based on non-negative matrix factorization to predict miRNA candidates associated with diseases. Functional similarity between miRNAs, similarities between diseases, and association information between miRNAs and diseases are fully utilized in our method. DMAPred not only considers the sparse nature of miRNA-disease association, but also deeply integrates the characteristics of miRNAs and diseases in low-dimensional space and the local topological information of miRNA nodes. Integrating the local topological information of a miRNA node can capture the association of the miRNA and its k most similar neighbors with similar diseases. Experimental results based on cross-validation are superior to several other methods, and the top ranking contains more real miRNA-disease associations. Case studies on breast, prostatic, and lung neoplasms were also carried out to demonstrate the ability of the DMAPred method to discover potential miRNAs.

## 2. Materials and Methods

Our aim was to predict potential miRNAs associated with diseases using the DMAPred method. First, a dual heterogeneous network composed of nodes, miRNAs, and diseases, was constructed to represent multiple relationships between miRNAs and diseases. Then, a new prediction model based on non-negative matrix factorization was applied to take into account the disease similarities, miRNA similarities, and associations between miRNAs and diseases. Finally, we obtained the final prediction scores for disease and miRNA by iterative optimization formula.

### 2.1. Dataset

Human miRNA-disease database (HMDD) has collected a great many associations between miRNAs and diseases that have been experimentally confirmed [34]. We got 5088 known associations from HMDD, which involved 490 miRNAs and 326 diseases. Disease terms were obtained from the National Library of Medicine (http://www.ncbi.nlm.nih.gov/mesh) to construct a directed acyclic graph (DAG) of diseases. The disease semantic similarity and phenotypic similarity were obtained from previous work [17]. 

### 2.2. Establishment of the miRNA-Disease Dual Heterogeneous Network

The dual heterogeneous network consisted of two types of nodes and three types of networks, which is the similarity network of miRNAs, the similarity network of diseases and the bipartite network between miRNAs and diseases.

**Figure 1:**
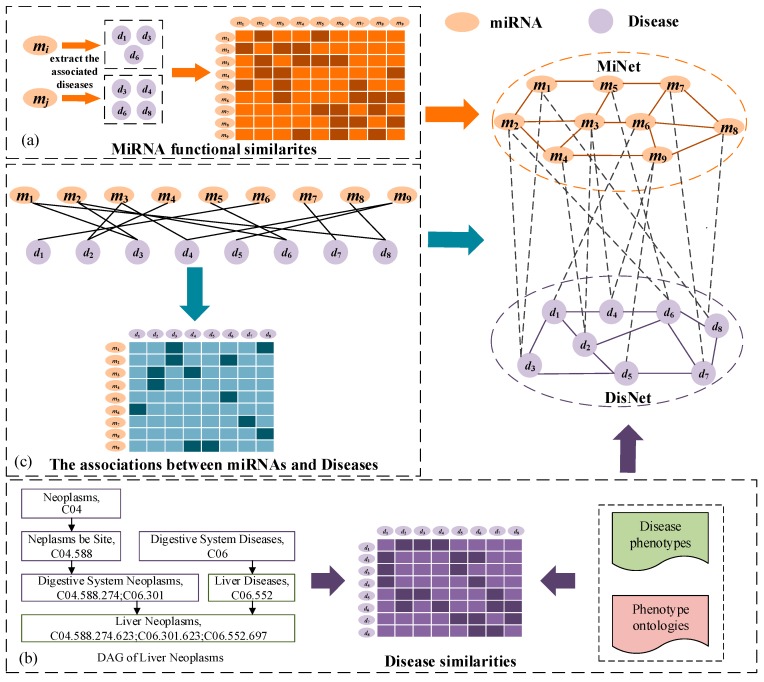
Construction and representation of a microRNA (miRNA)-disease heterogeneous network. (**a**) Calculate the miRNA similarity based on diseases associated with two miRNAs. (**b**) Construct the disease similarity by combining their disease phenotypes and phenotype ontologies. (**c**) Add edges between miRNAs and diseases.

Establishment of the miRNA network: The miRNA network (MiNet) was established on the similarity between miRNAs (Figure 1a). If two miRNAs were similar, we put an edge between two corresponding nodes. Every edge has a weight distributed between 0 and 1 to indicate the similarity of the nodes at both ends. Let matrix $M=\left[M_{ij}\right]\in R^{N_{m}\times N_{m}}$ denote the miRNAs network, where $M_{ij}$ represents the similarity between $i_{th}$ miRNA $m_{i}$ and $j_{th}$ miRNA $m_{j}$ and $N_{m}$ is the number of miRNAs. $R^{N_{m}\times N_{m}}$ is a real number set of dimensions $N_{m}\times \;N_{m}$. 

Two miRNAs that have similar functions are usually associated with similar diseases. Wang et al. [18] successfully calculated the similarity of miRNAs based on the similarity between the diseases that they were associated with. For example, miRNA $m_{i}$ is associated with a group of diseases $P_{i}=\left\{d_{3},d_{4},d_{6}\right\}$, miRNA $m_{j}$ is associated with a group of diseases $P_{j}=\left\{d_{1},d_{2},d_{4,}d_{8}\right\}$, the similarity between $m_{i}$ and $m_{j}$ is calculated based on the similarity of $P_{i}$ and $P_{j}$. The miRNA similarity that we used was calculated by the Wang’s method. 

Establishment of the disease network: The disease network is built on the similarity of diseases (Figure 1b). Every node in the disease network indicates a disease. We added an edge between two corresponding nodes when the two diseases were similar. The weight of every edge is the similarity between two diseases at both ends and is a positive number less than 1. The similarity between two diseases was estimated by disease semantic and phenotype [20]. The more common the disease semantic and phenotype, the more similar are the two diseases, and therefore the higher the possibility of associating with similar miRNAs. 

The matrix $D=\left[D_{ij}\right]\in R^{N_{d}\times N_{d}}$ represents the disease network, with $D_{ij}$ symbolizing the similarity between the $i_{th}$ disease and $j_{th}$ disease and the values of similarity are distributed between 0 and 1. The number of the diseases in disease network is $N_{d}$.

Establishment of the miRNA-disease bipartite network: A bipartite network that records the associations between diseases and miRNAs was constructed by adding the edge between two types of nodes (Figure 1c). This network is dissimilar from the other networks in that it contains two types of nodes and each edge connects two different types of nodes. If we identify from known association data that the disease $d_{j}$ is associated with the miRNA $m_{i}$, we add a side between corresponding nodes, and the weight of the edge is 1. Otherwise, when the associations between disease $d_{j}$ and the miRNA $m_{i}$ has not been discovered or does not exist, there is no edge between the nodes. 

The matrix $A=\left[A_{ij}\right]\in R^{N_{m}\times N_{d}}$ was constructed to record weight information for each edge of the bipartite network. The $i_{th}$ row of A is denoted as the associations between the miRNA $m_{i}$ and all the diseases, and the $j_{th}$ column of A is denoted as the associations between the disease $d_{j}$ and all the miRNAs. $A_{ij}$ is 1 when $m_{i}$ are observed to be associated with $d_{j}$ or 0 otherwise. 

### 2.3. miRNA-Disease Association Prediction Model

The proposed prediction model for predicting the potential miRNA-disease associations integrated multiple sources from three networks (namely, MiNet, DisNet, and MiDisNet). To make it easier to understand, we introduced a matrix $U=\left[U_{ij}\right]\in R^{N_{m}\times N_{d}}$. The matrix U is used to describe the scores of the association possibility between $N_{m}$ miRNAs and $N_{d}$ diseases, where $U_{ij}$ is a non-negative number indicating the association possibility between $m_{i}$ and $d_{j}$.

Modeling miRNA similarities: Three types of connections in MiDisNet can be used to construct the prediction model. The first type is the similarities between miRNAs in MiNet. Matrix M describes the miRNA similarities, where each row corresponds to the similarity between a miRNA and other miRNAs. For example, the $i_{th}$ row of M is denoted as the similarity between $m_{i}$ and all the other miRNAs. Data representation often has a large impact on the performance of the model. Projecting high-dimensional information into low-dimensional space contributes to the reduction of the original redundant information, thereby obtaining more dense and low-dimensional feature representations of the data. Therefore, we projected miRNA similarities in low-dimensional space by non-negative matrix factorization. Suppose $M=\left[M_{1},M_{2},\cdots M_{N_{m}}\right]\in R^{N_{m}\times N_{m}}$ is the non-negative $N_{m}$ data represents, where $M_{i}$ is the $i_{th}$ column of M and represents the $N_{m}$-dimensional original feature representation of the $i_{th}$ miRNA. Let $W=R^{N_{m}\times k}$ and $H=R^{k\times N_{m}}$ be the base matrix and the new representations of data in terms of the basis W and *k* is the dimension we require:

(1) M≈WH.

The result of *W* and *H* can well approximate the original matrix. Thus, we aimed to minimize the following objective function,

(2) $$\min{||M-WH||}_{F}^{2}\;,$$

where $\|\cdot {\|}_{F}$ is the Frobenius norm of the matrix. 

Modeling disease similarities: The second type of connection is similarities between diseases. The $j_{th}$ column of D represents the similarities between $d_{j}$ and all the diseases. We also projected disease similarities into low dimensional space similarly to the miRNAs to receive new representation of the diseases.

Suppose $D=\left[D_{1},D_{2},\ldots ,D_{N_{d}}\right]\in R^{N_{d}\times N_{d}}$ is the non-negative $N_{d}$ data matrix where each column is an original feature representation of a disease. Let $X\in R^{N_{d}\times k}$ be the base matrix and $C\in R^{k\times N_{d}}$ be the new data vector of diseases. The disease similarities are projected as follows,

(3) D≈XC.

Our aim was to find two matrices X and C whose product was closer to the original matrix. To better measure the matrix fitting, we added an item to the loss function,

(4) $$\min{||M-WH||}_{F}^{2}+\alpha {||D-XC||}_{F}^{2},$$

where α is a hyperparameter used to adjust the contribution of the disease similarity. 

Modeling the miRNA-disease associations: The third type of connection is the association between miRNAs and diseases. The miRNA-disease connections are recorded in matrix A in which each 1 represents an observed association. The matrix A was very sparse due to the small number of associations observed. Our model only considered the known associations in this situation. $Y=\left[Y_{ij}\right]\in R^{N_{m}\times N_{d}}$ was defined as an indicator matrix, and $Y_{ij}=1$ if $A_{ij}=1$ or 0 otherwise. The predicted scores for associations between $N_{m}$ miRNAs and $N_{d}$ diseases were recorded in U. The estimated association possibilities should be as close as possible to the known associations. As a result, we extended the objective function,

(5) $$\min{||M-WH||}_{F}^{2}+\alpha {||D-XC||}_{F}^{2}+\beta {||Y⊙\left(A-U\right)||}_{F}^{2},$$

where ⊙ is the multiplication of the corresponding elements of the matrix and *β* is a hyperparameter. 

Modeling the characteristics in the low-dimensional space: $H\in R^{k\times N_{m}}$ is the low-dimensional representation matrix of $N_{m}$ miRNAs, where the $i_{th}$ column is $m_{i}$. $C\in R^{k\times N_{d}}$ is the low-dimensional feature matrix of $N_{d}$ diseases, in which the $j_{th}$ column is $d_{j}$. $m_{i}\in R^{k}$ and $d_{j}\in R^{k}$ indicates the feature vectors of the $i_{th}$ miRNA and the $j_{th}$ disease, respectively. Our goal was to derive the association score between miRNA and disease by updating U in the model $U=H^{T}C$. Therefore, the loss function becomes,

(6) $$min{||M-WH||}_{F}^{2}+\alpha {||D-XC||}_{F}^{2}+\beta {||Y⊙(A-U)||}_{F}^{2}+\lambda {||U-H^{T}C||}_{F}^{2},$$

where λ is a hyperparameter.

Considering the sparse characteristic of associations: There are several diseases associated with a miRNA. Hence, the miRNA-disease associations have a sparse characteristic. We used 1-norm to ensure that the matrix U was sparse and added an item to the objective function as follows,

(7) $$min{||M-WH||}_{F}^{2}+\alpha {||D-XC||}_{F}^{2}+\beta {||Y⊙(A-U)||}_{F}^{2}+\lambda {||U-H^{T}C||}_{F}^{2},+\delta {||U||}_{1}.$$

Therefore, the non-zero elements in the matrix U were sparse. 

Modeling local topological information of the miRNAs: A miRNA and its k neighbors are usually associated with similar diseases. First, a graph model S was constructed, based on the similar properties of miRNAs. Each element in S was calculated according to the following formula,

(8) $$S_{jl}=\{\begin{array}{cc} 1 & {\mathrm{if}\;m}_{l}{\;\mathrm{is}\;\mathrm{the}\;k-\mathrm{nearest}\;\mathrm{neighbor}\;\mathrm{of}\;m}_{j}\\ 0 & \mathrm{otherwise}, \end{array}.$$

$u_{j}$ and $u_{l}$ are the associations between miRNA $m_{j}$ and $m_{l}$ and all the miRNAs, respectively. Set $S_{jl}$ to 1 when $m_{l}$ is the k-nearest neighbor of $m_{j}$. Thus, $u_{j}$ and $u_{l}$ should be as consistent as possible. Then, the finally loss function becomes,

(9) $$\begin{array}{c} min{||M-WH||}_{F}^{2}+\alpha {||D-XC||}_{F}^{2}+\beta {||Y⊙A-U||}_{F}^{2}\\ +\lambda {||U-H^{T}C||}_{F}^{2},+\delta {||U||}_{1}+\frac{1}{2}\eta \sum_{j,l=1}^{N}{||u_{j}-u_{l}||}^{2}S_{jl}, \end{array}$$

where ||⋅|| is the 2-norm; δ and η measure the contribution of the corresponding item in the formula. 

### 2.4. Optimization

The objective Function (7) is represented by *F*, which is a non-convex function. Therefore, it cannot guarantee direct global optimal solution. We proposed an iterative method to optimize the objective Function (7), and divide the problem of solving the objective function F into five sub-problems about the matrix U, W, H, X, and C. Then, the local optimal solution was found for each of the five sub-problems to obtain the global optimal solution. According to the conversion relationship between the trace property and the Frobenius norm of the matrix, F can be written as following, 

(10) $$\begin{array}{c} F=Tr\left(AA^{T}-AU^{T}-UA^{T}+UU^{T}\right)+\alpha Tr\left(MM^{T}-WHM^{T}-MH^{T}W^{T}+WHH^{T}W^{T}\right)+\beta Tr\\ \left(DD^{T}-XCD^{T}-DC^{T}X^{T}+XCC^{T}X^{T}\right)+\delta {||U||}_{1}+\lambda Tr(UU^{T}-UC^{T}H-H^{T}CU^{T}+\\ H^{T}CC^{T}H)+\delta B+\eta \mathrm{Tr}\left(\left(V-S\right)U+{\left(V-S\right)}^{T}U\right). \end{array}$$

Tr(⋅) represents the trace of the matrix, which is the sum of the values on the main diagonal of the matrix. Here $V\in R^{N_{m}\times N_{m}}$ is a diagonal matrix where each element is defined as $V_{ii}=\sum_{k=0}^{N_{m}-1}S_{ik}\left(i=0,1,2,\cdots ,N_{m}-1\right)$. $B\in R^{N_{m}\times N_{d}}$ is a matrix in which each element is 1. 

U sub-problem: When updating U, the other four matrices W, H, X, and C were fixed. The sub-problem about U can be written as,

(11) $$F\left(U\right)=Tr\left(AA^{T}-AU^{T}-UA^{T}+UU^{T}\right)+\delta {||U||}_{1}+\lambda Tr\left(UU^{T}-UC^{T}H-H^{T}CU^{T}+\;H^{T}CC^{T}H\right)+\delta B+\eta Tr(\left(V-S\right)U+\left(V-S{)}^{T}U\right).$$

The derivative of the objective function for *U* was set to 0. Then there is:

(12) $$\frac{\partial F}{\partial U}=2U-2A+2\lambda \left(U-H^{T}C\right)+2\eta \left[\left(V-S\right)U\right]=0.$$

After multiplying both sides of the above equation by $U_{ij}$, the following formula was obtained,

(13) $${\left(2U-2A+2\lambda \left(U-H^{T}C\right)+2\eta \left[\left(V-S\right)U\right]\right)}_{ij}U_{ij}=0.$$

Finally, according to the gradient descent algorithm, we obtained the local optimal solution of U in the current situation. Updated *U* was as follows,

(14) $$U_{ij}^{new}\leftarrow U_{ij}\cdot \frac{{\left(2A+2\lambda H^{T}C+2\eta S_{M}U\right)}_{ij}}{{\left(2U+2\lambda U+2\eta V_{M}U\right)}_{ij}}.$$

H sub-problem: When the matrices U, W, X, and C are fixed, the sub-problem about H can be written as,

(15) $$\begin{array}{c} F\left(H\right)=\alpha Tr\left(MM^{T}-WHM^{T}-MH^{T}W^{T}+WHH^{T}W^{T}\right)+\\ \lambda Tr\left(UU^{T}-UC^{T}H-H^{T}CU^{T}+H^{T}CC^{T}H\right). \end{array}$$

Let the derivative of the objective function *F* to *H* be 0. Then we have:

(16) $$\frac{\partial F}{\partial H}=2\alpha W^{T}WH-2\alpha W^{T}M+2\lambda CC^{T}H-2\lambda CU^{T}=0.$$

Multiply both sides of the equation by *A*, we obtained:

(17) $$\left(2\alpha W^{T}WH-2\alpha W^{T}M+2\lambda CC^{T}H-2\lambda CU^{T}\right)H_{ij}=0.$$

Finally, we got the update formula of matrix H by gradient descent method as follows,

(18) $$H_{ij}^{new}\leftarrow H_{ij}\cdot \frac{(2\alpha W^{T}M+2\lambda CU^{T}{)}_{ij}}{{\left(2\alpha W^{T}WH+2\lambda CC^{T}H\right)}_{ij}}.$$

Then, the same method was used to find the formula to update W, X, and C. The remaining four matrices were fixed when updating a matrix. We obtained three optimization formulas for the other matrices,

(19) $$W_{ij}^{new}\leftarrow W_{ij}\cdot \frac{{\left(2MH^{T}\right)}_{ij}}{{\left(2WHH^{T}\right)}_{ij}},$$

(20) $$X_{ij}^{new}\leftarrow X_{ij}\cdot \frac{{\left(2DC^{T}\right)}_{ij}}{{\left(2XCC^{T}\right)}_{ij}},$$

(21) $$C_{ij}^{new}\leftarrow C_{ij}\cdot \frac{{\left(2\alpha X^{T}D+2\lambda HU\right)}_{ij}}{{\left(2\alpha X^{T}XC+2\lambda HH^{T}C\right)}_{ij}}$$

**Figure 2:**
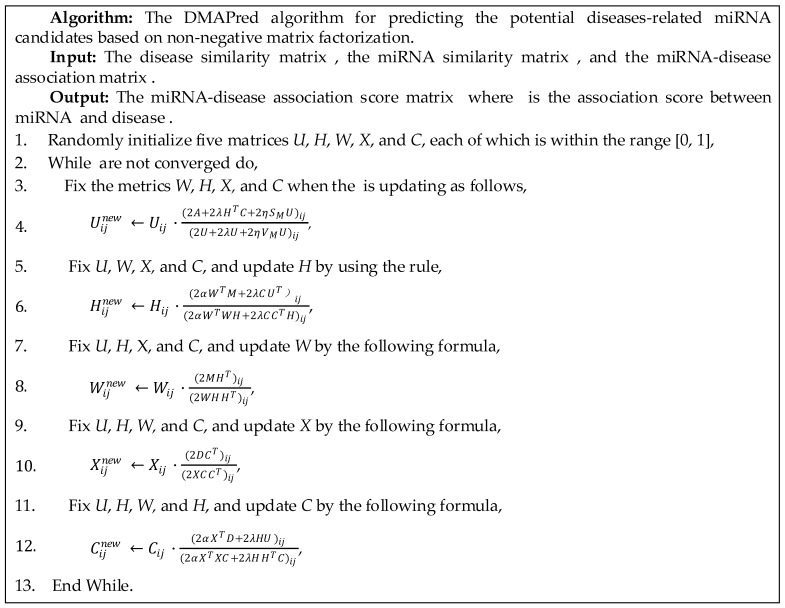
Iterative algorithms for predicting the potential diseases-related miRNA candidates.

The $j_{th}$ column of the final matrix U represents the association scores between the *j_th_* disease and all miRNAs (Figure 2). The miRNAs associated with the disease were not found to be sorted according to the association score in U. In the ordered list, the higher the position of the miRNAs based association score, the more likely it is to be a potential miRNA associated with the disease.

## 3. Performance Evaluation and Analysis

### 3.1. Performance Evaluation

To evaluate the algorithm performance, we performed fivefold cross validation. In the fivefold cross validation, all known associations between miRNAs and drugs were randomly divided into five subsets. Each time, we used four subsets to train the model, and the remaining one was used as a test set. For a disease $d_{j}$, miRNAs associated with disease $d_{j}$ are considered positive, and unlabeled miRNAs that were not associated with disease, were considered negative. The higher the positive samples order, the better the prediction performance of the algorithm.

Given a threshold θ, if the associated prediction score was greater than θ, it was judged as a positive example, otherwise it will be judged as negative. The true positive rate (*TPR*) and false positive rate (*FPR*) according to the following formulas,

(22) $$TPR=\frac{TP}{TP+FN}\;,\;FPR=\frac{FP}{TN+FP},$$

where TP and TN represent the number of positive and negative examples, respectively. FN and FP represent the number of predicted errors in positive and negative examples. The *TPR* and FPR at different thresholds can be used to plot the Receiver Operating Characteristic (ROC) curve. The area under the ROC curve (AUC) can reflect the comprehensive prediction performance of the algorithm. The larger the AUC, the better the comprehensive prediction performance.

In the miRNA-disease association data, the number of known associations was much smaller than the unknown association, which created a serious imbalance between the positive and negative samples. In the case of positive and negative imbalances, precision and recall are more suitable for measuring the performance of the method. The precision *P* and the recall *R* are defined as,

(23) $$P=\frac{TP}{TP+FP}\;,\;R=\frac{TP}{TP+FN}.$$

*P* represents how many of the samples predicted to be positive are correct, and *R* indicates how much of the positive examples are correctly identified by the model. We calculated precision and recall at different thresholds, and used the precision as the vertical axis and the recall as the horizontal axis to obtain the P–R curve. The area under the PR curve (AUPR) indicates the predictive performance of the model in certain aspects. The larger the AUPR, the better the predictive ability of the model.

In the process of biological research, biologists often select the top miRNA candidates for further biological experiments. To identify how many of the positive examples among the top candidates are important for biological research, we computed the recall rate within top *k* to measure the performance of the prediction model.

### 3.2. Comparison with Other Methods

To confirm that the proposed method has a superior performance in predicting potential miRNA candidates associated with diseases, we compared DMAPred with Liu’s method [22], DMPred [35], PBMDA [29], GSTRW [36], and BNPDMA [37], which are state-of-the-art prediction methods for miRNA-disease associations. Liu et al. integrated the similarities and associations between miRNAs and diseases to propose a method of random walks with a restart in a heterogeneous miRNA-disease network to predict the association score between a miRNA and a disease. You et al. proposed a method, PBMDA, based on the path to predict the likelihood of a miRNA association with a disease. This method not only integrates the similarity of miRNA functions and the semantic similarity of diseases, but also considers the similarity of the Gaussian interaction spectrum between miRNAs and diseases. Xuan et al. proposed DMPred, based on non-negative matrix factorization, to predict the associations between miRNAs and diseases taking into account the sparse nature of miRNA disease associations. Chen et al. proposed a method, called GSTRW, that calculates the global similarity of a network and predicts the association between a miRNA and a disease by performing random walks in miRNA and disease similarity networks, respectively. BNPDMA uses a bipartite recommendation algorithm to predict potential disease-associated miRNAs by assigning bias ratings to the associations between miRNAs and diseases.

Several hyperparameters in the objective function might impact the performance of the proposed algorithm. By enumerating the sensitivity of each parameter, we selected the values of the parameters α, β, λ, δ, η from {0.1, 0.4, 0.8, 1,4, 8}. The contribution of each parameter to the algorithm was measured by varying each parameter to compare the AUC values. Finally, we established the parameters as α=0.1, β=0.1, γ=0.1, and δ=1, η=0.4 by comparing the AUC values for the different parameters. 

**Figure 3:**
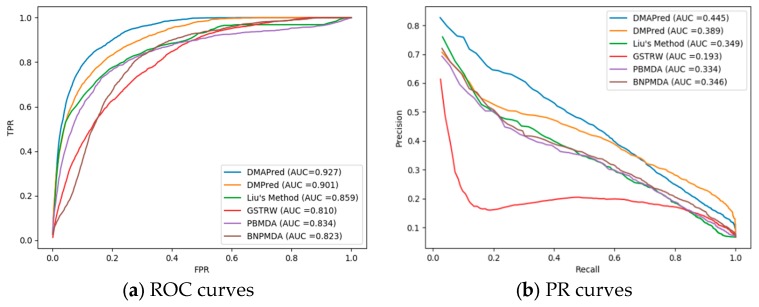
Two types of curves for evaluating the predicting performance of DMAPred and other five methods. (**a**) the Receiver Operating Characteristic (ROC) curves and area under the receiver operating characteristic curve (AUC) values of DMAPred and other five methods; and (**b**) precision–recall (PR) curves and area under the PR curve (AUPR) values of DMAPred and other five methods.

The predictive performances of the proposed method and Liu’s method, DMPred, GSTRW, PBMDA, and BNPMDA for all the diseases were compared based on different evaluation criteria. Figure 3a shows the average ROC curves for DMAPred and the other five methods for the 326 diseases. The average AUC values obtained with DMAPred, Liu’s method, DMPred, GSTRW, PBMDA, and BNPDMA were 0.927, 0.859, 0.901, 0.810, 0.834, and 0.823, respectively. 

The proposed method, DMAPred, achieved the best performance, with the average AUC value being higher than those obtained using the other five methods by 6.8%, 2.6%, 11.7%, 9.3%, and 10.4%, respectively. The faster the TPR values grow versus FPR values, the larger the AUC value for the corresponding ROC curve is. However, the growth rate of TPR is affected by the predicted association scores of positive samples. The larger the predicted score of the positive samples is, the closer our prediction results are to the actual values and the faster the TPR grows. Among the five other methods, the performance of the DMPred method was the second best. This method is based on the matrix factorization, similar to our method, although the calculation of disease similarity and miRNA similarity takes into account factors different from ours. Liu’s method was a little worse than other methods, the main reason being that the calculation of similarity between miRNAs is indirectly measured by genes and LncRNA, and does not take into account the direct relationship between miRNA and disease. The GSTRW method was the worst of the four methods probably because it uses a two-layer random walk. We also list the AUCs for 15 well-characterized diseases associated with at least 80 miRNAs (Table 1). DMAPred achieved the best predictive performance for 10 of the 15 well-characterized diseases.

The PR curve reacts better than the ROC to reflect the predictive performance of different methods when the positive and negative examples in the data set are unbalanced. Figure 3b shows the PR curve for DMAPred and the other five methods with an average AUPR of 0.445, 0.389, 0.349, 0.193, 0.334, and 0.346 for 326 diseases. The performance of DMAPred was evaluated as the best and GSTRW was the worst. DMAPred was 5.6%, 9.6%, 25.2%, 11.3%, and 9.9% higher than the other methods. Table 2 shows the AUPR values of DMAPred and the other five methods for 15 diseases. DMAPred achieved best performance for 10 among the 15 diseases. 

**Figure 4:**
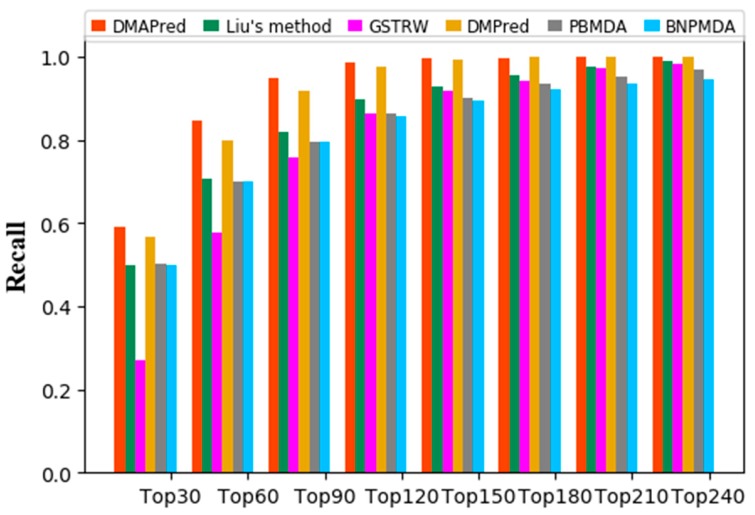
Average recalls of all the diseases at different top *k*.

The larger the recall value of top *k* in the ranked list indicates that more positive examples in the top k miRNA candidates are identified (Figure 4). DMAPred performed better than all other methods, with 59.19% in the top 30 candidates, 84.67% in the top 60, and 94.88% in the top 90. DMPred’s performance achieved the second best, with 56.76% in the top 30 candidates, 79.82% in the top 60, and 91.68% in the top 90. Liu’s method was slightly worse, with 50.01% in the top 30 candidates, 70.52% in the top 60, and 81.84% in the top 90. The performance of PBMDA showed with 50.11% in the top 30 candidates, 70.14% in the top 60, and 79.49% in the top 90. GSTRW was the worst, with recalls of 26.90%, 57.79%, and 75.89%, respectively.

In addition, we conducted a *t*-test to further prove that our method was superior to others in AUC and AUPR. All paired *t*-test results less than 0.05 means that our method was better than the other methods (Table 3). 

### 3.3. Case Studies on Breast Neoplasms, Prostatic Neoplasms, and Lung Neoplasms

To further demonstrate our approach in identifying potential disease-related miRNAs, we conducted case studies for the top 50 candidates for breast neoplasms, prostate neoplasms, and lung neoplasms. The top 50 candidates related to breast neoplasms are listed for detailed analysis and verification (Table 4). 

The databases involved were dbDEMC [44] and PhenomiR [45]. The dbDEMC database contained 807 miRNAs with significant abnormal expression levels in human cancer and has an online public database. The PhenomiR database contains miRNA expression information that is differentially regulated during disease, and its data was extracted from more than 365 scientific articles. Using the dbDEMC database, we found 42 of the 50 candidates were up-regulated or down-regulated in breast neoplasms. Thirty-five of the 50 miRNA candidates were included in PhenomiR. The remaining five miRNAs labeled ‘Literature’ were supported by relevant research literatures. 

The top 50 candidates associated with prostate neoplasms are listed in supplementary table ST1. Abnormal expression of 39 candidates in prostate neoplasms was included in the dbDEMC2 database and 36 candidates were included in the PhenomiR database. Three candidates marked ‘Literature’ means that it was supported by the relevant literatures. There were several miRNAs labeled ‘Unconfirm’, which were associated with prostate neoplasms without a relevant database or literature support.

The top 50 candidates associated with lung neoplasms are shown in supplementary table ST2. Abnormal expression of 29 candidates with up-regulation or down-regulation in lung neoplasms was recorded in the dbDEMC2 database, and seven candidates were confirmed by relevant literature. The PhenomiR database included abnormal regulation of 17 candidates in the lung neoplasms. Analysis of breast neoplasms, prostate neoplasms, and lung neoplasms predictions further demonstrates the ability of our methods to predict disease-associated miRNAs. 

## 4. Conclusions

The method based on non-negative matrix factorization, DMAPred, was developed to predict potential miRNAs associated with diseases. DMAPred captures the internal relationships of miRNAs and diseases, including miRNA similarities and disease similarities, and the relationship between miRNAs and diseases, i.e., miRNA-disease associations. Moreover, local topological information for each node in the miRNA network and dense features of miRNAs and diseases in low-dimensional space also contributes for screening of potential disease miRNA candidates. The objective problem was divided into five sub-problems. An iterative algorithm was developed to obtain the final miRNA-disease association scores that could be used to rank the candidate miRNAs for each disease. In our experiment, DMAPred was found to be superior to several other methods, with regard to both AUCs and AUPRs. In addition, DMAPred can help biologists to find candidates they are interested in because the top ranking list contains more true miRNA-disease associations. Case studies on three diseases confirmed that DMAPred is able to discover potential miRNA candidates associated with specific disease.

## Figures and Tables

**Table 1:** AUC values of five methods for all the diseases and 15 common diseases.

Diseases Name	AUC					
	DMAPred	GSTRW	DMPred	PBMDA	Liu’s Method	BNPMDA
Breast neoplasms	**0.966**	0.822	0.938	0.852	0.863	0.905
Hepatocellular carcinoma	**0.957**	0.779	0.900	0.803	0.845	0.853
Renal cell carcinoma	**0.926**	0.816	0.903	0.813	0.832	0.845
Squamous cell carcinoma	**0.942**	0.817	0.908	0.881	0.890	0.877
Colorectal neoplasms	**0.895**	0.737	0.842	0.826	0.857	0.801
Glioblastoma	**0.928**	0.814	0.904	0.803	0.842	0.817
Heart failure	0.965	0.817	**0.987**	0.791	0.828	0.891
Acute myeloid leukemia	**0.967**	0.788	0.890	0.844	0.874	0.845
Lung neoplasms	**0.973**	0.791	0.948	0.905	0.920	0.912
Melanoma	0.907	0.789	**0.913**	0.836	0.860	0.889
Ovarian neoplasms	**0.939**	0.830	0.929	0.889	0.897	0.725
Pancreatic neoplasms	**0.933**	0.838	0.916	0.891	0.904	0.829
Prostatic neoplasms	**0.958**	0.822	0.951	0.843	0.855	0.894
Stomach neoplasms	**0.935**	0.762	0.908	0.821	0.836	0.784
Urinary bladder neoplasms	0.860	0.816	**0.919**	0.854	0.865	0.901
Average AUC for the 326 diseases	**0.927**	0.810	0.901	0.834	0.859	0.823

Bold values indicate the higher AUCs.

**Table 2:** AUPR values of five methods for all the diseases and 15 common diseases.

Disease Name	AUPR					
	DMAPred	Liu’s Method	GSTRW	DMPred	PBMDA	BNPMDA
Breast neoplasms	**0.761**	0.573	0.322	0.699	0.574	0.254
Hepatocellular carcinoma	**0.719**	0.498	0.279	0.501	0.454	0.618
Renal cell carcinoma	**0.485**	0.186	0.150	0.293	0.181	0.334
Squamous cell carcinoma	**0.299**	0.208	0.109	0.213	0.211	0.214
Colorectal neoplasms	0.340	**0.371**	0.141	0.186	0.367	0.197
Glioblastoma	**0.517**	0.243	0.151	0.219	0.217	0.227
Heart failure	**0.786**	0.189	0.191	0.700	0.168	0.178
Acute myeloid leukemia	**0.317**	0.236	0.140	0.211	0.191	0.190
Lung neoplasms	**0.740**	0.503	0.147	0.511	0.537	0.547
Melanoma	0.342	**0.397**	0.171	0.389	0.363	0.334
Ovarian neoplasms	**0.441**	0.361	0.169	0.404	0.361	0.357
Pancreatic neoplasms	0.303	0.354	0.137	0.329	**0.364**	0.357
Prostatic neoplasms	**0.532**	0.264	0.166	0.463	0.282	0.345
Stomach neoplasms	**0.469**	0.346	0.220	0.446	0.344	0.284
Urinary bladder neoplasms	0.118	0.280	0.163	**0.315**	0.252	0.242
Average AUPR for the 326 diseases	**0.445**	0.349	0.193	0.389	0.334	0.346

Bold values indicate the higher AUPRs.

**Table 3:** Comparison of different methods based on AUC and AUPR with a paired *t*-test.

	DMPred	Liu’s Method	GSTRW	PBMDA	BNPMDA
***p*-value of AUCs**	0.00247	5.0135 × 10^−7^	2.4835 × 10^−9^	2.3143 × 10^−6^	9.5824 × 10^−6^
***p*-value of AUPRs**	0.00168	0.00199	3.6475 × 10^−6^	0.00289	0.00182

**Table 4:** The top 50 candidates related to breast neoplasms.

Rank	MiRNA Name	Description	Rank	MiRNA Name	Description
1	hsa-mir-15b	dbDEMC2,PhenomiR	26	hsa-mir-184	dbDEMC2,PhenomiR
2	hsa-mir-142	PhenomiR	27	hsa-mir-363	dbDEMC2
3	hsa-mir-192	PhenomiR	28	hsa-mir-30e	PhenomiR
4	hsa-mir-378a	Literature [38]	29	hsa-mir-208a	dbDEMC2,PhenomiR
5	hsa-mir-106a	dbDEMC2,PhenomiR	30	hsa-mir-449b	dbDEMC2
6	hsa-mir-99a	dbDEMC2,PhenomiR	31	hsa-mir-491	PhenomiR
7	hsa-mir-130a	dbDEMC2,PhenomiR	32	hsa-mir-494	dbDEMC2,PhenomiR
8	hsa-mir-150	dbDEMC2,PhenomiR	33	hsa-mir-186	dbDEMC2,PhenomiR
9	hsa-mir-196b	dbDEMC2,PhenomiR	34	hsa-mir-362	Literature [39]
10	hsa-mir-130b	dbDEMC2,PhenomiR	35	hsa-mir-424	dbDEMC2,PhenomiR
11	hsa-mir-98	dbDEMC2,PhenomiR	36	hsa-mir-370	dbDEMC2,PhenomiR
12	hsa-mir-1266	dbDEMC2	37	hsa-mir-542	Literature [40]
13	hsa-mir-92b	dbDEMC2	38	hsa-mir-32	dbDEMC2,PhenomiR
14	hsa-mir-372	dbDEMC2,PhenomiR	39	hsa-mir-181d	dbDEMC2,PhenomiR
15	hsa-mir-138	dbDEMC2,PhenomiR	40	hsa-mir-483	PhenomiR
16	hsa-mir-574	Literature [41,42]	41	hsa-mir-302e	dbDEMC2
17	hsa-mir-144	dbDEMC2,PhenomiR	42	hsa-mir-302f	dbDEMC2
18	hsa-mir-28	dbDEMC2,PhenomiR	43	hsa-mir-208b	dbDEMC2
19	hsa-mir-212	dbDEMC2,PhenomiR	44	hsa-mir-134d	dbDEMC2
20	hsa-mir-181c	dbDEMC2,PhenomiR	45	hsa-mir-330	dbDEMC2,PhenomiR
21	hsa-mir-371a	Literature [43]	46	hsa-mir-381	dbDEMC2,PhenomiR
22	hsa-mir-449a	dbDEMC2,PhenomiR	47	hsa-mir-198	dbDEMC2,PhenomiR
23	hsa-mir-185	dbDEMC2,PhenomiR	48	hsa-mir-548a	dbDEMC2
24	hsa-mir-211	dbDEMC2,PhenomiR	49	hsa-mir-154	dbDEMC2,PhenomiR
25	hsa-mir-99b	dbDEMC2,PhenomiR	50	hsa-mir-503	dbDEMC2

## Supplementary Materials

The following are available online at https://www.mdpi.com/2073-4425/10/9/685/s1. Table ST1: The top 50 candidates for prostatic neoplasms. Table ST2: The top 50 candidates for lung neoplasms. Table ST3: The top 50 potential candidates for 326 diseases. Table ST4: The specific hyperparameters of the five methods and their values.
