# Supplementary material for: Predicting miRNA-Disease Associations by Incorporating Projections in Low-Dimensional Space and Local Topological Information
Source: Genes (Basel). 2019 Sep 6;10(9):685. doi: 10.3390/genes10090685 (PMC6770973; doi:10.3390/genes10090685)
Supplement: Supplementary file 1 [file genes-10-00685-s001.zip › ST1_The top 50 candidates for Prostatic cancer.docx]

**ST1.** The top 50 candidates related to prostatic neoplasms

| Rank | MiRNA name | Description | | Rank | MiRNA name | Description |
| --- | --- | --- | --- | --- | --- | --- |
| 1 | hsa-mir-29c | dbDEMC2,PhenomiR | 26 | | hsa-mir-28 | dbDEMC2,PhenomiR |
| 2 | hsa-mir-206 | dbDEMC2,PhenomiR | 27 | | hsa-mir-9 | Literature[1] |
| 3 | hsa-mir-944 | dbDEMC2 | 28 | | hsa-mir-181a | dbDEMC2 |
| 4 | hsa-mir-1229 | Unconfirm | 29 | | hsa-mir-30a | PhenomiR |
| 5 | hsa-mir-1227 | dbDEMC2 | 30 | | hsa-let-7f | dbDEMC2 |
| 6 | hsa-mir-103a | Literature[2] | 31 | | hsa-mir-10a | dbDEMC2,PhenomiR |
| 7 | hsa-mir-499a | dbDEMC2 | 32 | | hsa-mir-449b | dbDEMC2 |
| 8 | hsa-mir-142 | PhenomiR | 33 | | hsa-mir-572 | dbDEMC2 |
| 9 | hsa-mir-196a | dbDEMC2 | 34 | | hsa-mir-181c | dbDEMC2,PhenomiR |
| 10 | hsa-mir-155 | dbDEMC2,PhenomiR | 35 | | hsa-mir-197 | dbDEMC2,PhenomiR |
| 11 | hsa-mir-199b | dbDEMC2,PhenomiR | 36 | | hsa-mir-24 | dbDEMC2 |
| 12 | hsa-mir-2861 | Unconfirm | 37 | | hsa-mir-497 | dbDEMC2,PhenomiR |
| 13 | hsa-mir-3196 | Unconfirm | 38 | | hsa-mir-542 | Literature[3] |
| 14 | hsa-mir-192 | dbDEMC2 | 39 | | hsa-mir-19a | dbDEMC2,PhenomiR |
| 15 | hsa-mir-338 | dbDEMC2,PhenomiR | 40 | | hsa-mir-571 | dbDEMC2 |
| 16 | hsa-mir-150 | dbDEMC2,PhenomiR | 41 | | hsa-mir-18a | dbDEMC2,PhenomiR |
| 17 | hsa-mir-210 | dbDEMC2,PhenomiR | 42 | | hsa-mir-124a | Literature[4] |
| 18 | hsa-mir-30b | PhenomiR | 43 | | hsa-mir-1233 | Literature[5] |
| 19 | hsa-mir-7 | dbDEMC2 | 44 | | hsa-mir-1285 | dbDEMC2 |
| 20 | hsa-mir-219 | Literature[6] | 45 | | hsa-mir-494 | dbDEMC2 |
| 21 | hsa-mir-429 | Literature[7] | 46 | | hsa-let-7g | dbDEMC2 |
| 22 | hsa-mir-138 | Literature[8] | 47 | | hsa-mir-144 | dbDEMC2,PhenomiR |
| 23 | hsa-mir-128 | Literature[9] | 48 | | hsa-let-7e | dbDEMC2,PhenomiR |
| 24 | hsa-mir-451a | dbDEMC2 | 49 | | hsa-let-7i | dbDEMC2,PhenomiR |
| 25 | hsa-mir-19b | dbDEMC2 | 50 | | hsa-mir-372 | dbDEMC2,PhenomiR |

References

1. Chen, L.-t.; Xu, S.-d.; Xu, H.; Zhang, J.-f.; Ning, J.-f.; Wang, S.-f.J.M.O. MicroRNA-378 is associated with non-small cell lung cancer brain metastasis by promoting cell migration, invasion and tumor angiogenesis. **2012**, *29*, 1673-1680.

2. Fu, X.; Zhang, W.; Su, Y.; Lu, L.; Wang, D.; Wang, H.J.T.P. MicroRNA‐103 suppresses tumor cell proliferation by targeting PDCD10 in prostate cancer. **2016**, *76*, 543-551.

3. Rabiau, N.; Trraf, H.-K.; Adjakly, M.; Bosviel, R.; Guy, L.; Fontana, L.; Bignon, Y.-J.; Bernard-Gallon, D.J.J.i.v. miRNAs differentially expressed in prostate cancer cell lines after soy treatment. **2011**, *25*, 917-921.

4. Shi, X.-B.; Xue, L.; Ma, A.-H.; Tepper, C.G.; Gandour-Edwards, R.; Kung, H.-J.; deVere White, R.W.J.O. Tumor suppressive miR-124 targets androgen receptor and inhibits proliferation of prostate cancer cells. **2013**, *32*, 4130.

5. Wulfken, L.M.; Moritz, R.; Ohlmann, C.; Holdenrieder, S.; Jung, V.; Becker, F.; Herrmann, E.; Walgenbach-Brünagel, G.; von Ruecker, A.; Müller, S.C.J.P.o. MicroRNAs in renal cell carcinoma: diagnostic implications of serum miR-1233 levels. **2011**, *6*, e25787.

6. Ren, Q.; Liang, J.; Wei, J.; Basturk, O.; Wang, J.; Daniels, G.; Gellert, L.L.; Li, Y.; Shen, Y.; Osman, I.J.A.j.o.t.r. Epithelial and stromal expression of miRNAs during prostate cancer progression. **2014**, *6*, 329.

7. Ouyang, Y.; Gao, P.; Zhu, B.; Chen, X.; Lin, F.; Wang, X.; Wei, J.; Zhang, H.J.M.m.r. Downregulation of microRNA-429 inhibits cell proliferation by targeting p27Kip1 in human prostate cancer cells. **2015**, *11*, 1435-1441.

8. Walter, B.A.; Valera, V.A.; Pinto, P.A.; Merino, M.J.J.J.o.C. Comprehensive microRNA profiling of prostate cancer. **2013**, *4*, 350.

9. Jin, M.; Zhang, T.; Liu, C.; Badeaux, M.A.; Liu, B.; Liu, R.; Jeter, C.; Chen, X.; Vlassov, A.V.; Tang, D.G.J.C.r. miRNA-128 suppresses prostate cancer by inhibiting BMI-1 to inhibit tumor-initiating cells. **2014**, *74*, 4183-4195.
