# Supplementary material for: Predicting miRNA-Disease Associations by Incorporating Projections in Low-Dimensional Space and Local Topological Information
Source: Genes (Basel). 2019 Sep 6;10(9):685. doi: 10.3390/genes10090685 (PMC6770973; doi:10.3390/genes10090685)
Supplement: Supplementary file 1 [file genes-10-00685-s001.zip › ST2_The top 50 candidates for Lung cancer.docx]

**ST 2.** The top 50 candidates related to lung neoplasms

| Rank | MiRNA name | Description | | Rank | MiRNA name | Description |
| --- | --- | --- | --- | --- | --- | --- |
| 1 | hsa-mir-193b | dbDEMC2,PhenomiR | 26 | | hsa-mir-429 | dbDEMC2 |
| 2 | hsa-mir-378a | Literature[1] | 27 | | hsa-mir-122 | dbDEMC2,PhenomiR |
| 3 | hsa-mir-151a | Literature[2] | 28 | | hsa-mir-92b | dbDEMC2,PhenomiR |
| 4 | hsa-mir-16 | dbDEMC2,PhenomiR | 29 | | hsa-mir-449b | dbDEMC2,PhenomiR |
| 5 | hsa-mir-15a | PhenomiR | 30 | | hsa-mir-184 | dbDEMC2,PhenomiR dbDEMC2,PhenomiR dbDEMC2,PhenomiR |
| 6 | hsa-mir-106b | dbDEMC2,PhenomiR dbDEMC2,PhenomiR iR | 31 | | hsa-mir-99b | dbDEMC2,PhenomiR |
| 7 | hsa-mir-708 | dbDEMC2 | 32 | | hsa-mir-23b | dbDEMC2,PhenomiR |
| 8 | hsa-mir-99a | dbDEMC2,PhenomiR | 33 | | hsa-mir-328 | dbDEMC2,PhenomiR |
| 9 | hsa-mir-149 | dbDEMC2,PhenomiR | 34 | | hsa-mir-372 | dbDEMC2,PhenomiR |
| 10 | hsa-mir-10a | dbDEMC2,PhenomiR | 35 | | hsa-mir-194 | dbDEMC2 |
| 11 | hsa-mir-196b | dbDEMC2,PhenomiR | 36 | | hsa-mir-625 | dbDEMC2 |
| 12 | hsa-mir-15b | dbDEMC2,PhenomiR | 37 | | hsa-mir-148b | dbDEMC2,PhenomiR |
| 13 | hsa-mir-130a | dbDEMC2,PhenomiR | 38 | | hsa-mir-320a | PhenomiR |
| 14 | hsa-mir-144 | dbDEMC2,PhenomiR | 39 | | hsa-mir-345 | dbDEMC2,PhenomiR |
| 15 | hsa-mir-451a | dbDEMC2 | 40 | | hsa-mir-362 | PhenomiR |
| 16 | hsa-mir-141 | dbDEMC2,PhenomiR dbDEMC2,PhenomiR C | 41 | | hsa-mir-491 | dbDEMC2 |
| 17 | hsa-mir-195 | dbDEMC2,PhenomiR | 42 | | hsa-mir-1236 | dbDEMC2 |
| 18 | hsa-mir-342 | dbDEMC2,PhenomiR | 43 | | hsa-mir-130b | dbDEMC2 |
| 19 | hsa-mir-296 | PhenomiR | 44 | | hsa-mir-151b | dbDEMC2 |
| 20 | hsa-mir-449a | dbDEMC2,PhenomiR | 45 | | hsa-mir-424 | dbDEMC2,PhenomiR |
| 21 | hsa-mir-208a | PhenomiR | 46 | | hsa-mir-370 | dbDEMC2,PhenomiR |
| 22 | hsa-mir-363 | PhenomiR | 47 | | hsa-mir-423 | PhenomiR |
| 23 | hsa-mir-28 | dbDEMC2,PhenomiR | 48 | | hsa-mir-208b | Literature[3] |
| 24 | hsa-mir-373 | dbDEMC2,PhenomiR | 49 | | hsa-mir-139 | dbDEMC2,PhenomiR |
| 25 | hsa-mir-99b | dbDEMC2,PhenomiR | 50 | | hsa-mir-302a | dbDEMC2,PhenomiR |

References

1. Chen, L.-t.; Xu, S.-d.; Xu, H.; Zhang, J.-f.; Ning, J.-f.; Wang, S.-f.J.M.O. MicroRNA-378 is associated with non-small cell lung cancer brain metastasis by promoting cell migration, invasion and tumor angiogenesis. **2012**, *29*, 1673-1680.

2. Wang, T.; Lv, M.; Shen, S.; Zhou, S.; Wang, P.; Chen, Y.; Liu, B.; Yu, L.; Hou, Y.J.P.o. Cell-free microRNA expression profiles in malignant effusion associated with patient survival in non-small cell lung cancer. **2012**, *7*, e43268.

3. Jin, J.; Deng, J.; Wang, F.; Xia, X.; Qiu, T.; Lu, W.; Li, X.; Zhang, H.; Gu, X.; Liu, Y.J.T.B. The expression and function of microRNA-203 in lung cancer. **2013**, *34*, 349-357.
